# Research on intelligent matching of students’ learning ability and healthcare job market demand based on industrial engineering expertise graph

**DOI:** 10.3389/frai.2025.1650095

**Published:** 2025-09-12

**Authors:** Yan Xiao, Lingtao Zeng, Jie Yang, Mini Han Wang, Zhiyuan Lin, Wei Li

**Affiliations:** ^1^Department of Mechanical Engineering, Chongqing University of Technology, Chongqing, China; ^2^Department of Medicine, Frontier Science Computing Center, Zhuhai Institute of Advanced Technology Chinese Academy of Sciences, The Chinese University of Hong Kong, Hong Kong, Hong Kong SAR, China; ^3^Department of Computer, Beijing Institute of Technology, Zhuhai Campus, Zhuhai, China

**Keywords:** expertise graph, industrial engineering, BERT–BiLSTM–GCN model, employment matching algorithm, intelligent matching

## Abstract

In China, there is a structural mismatch between the job market and student employment, characterized by “unfilled jobs” and “unqualified candidates,” particularly between the industrial engineering (IE) profession and the healthcare services sector. Expertise graphs are designed to identify the logical connections between academic disciplines and job market needs, linking students’ knowledge and skills with job requirements. This approach provides a systematic and visual alignment between students’ learning outcomes and job market demands, addressing the mismatch. However, current expertise graphs have not effectively captured the intrinsic connection between students’ learning abilities and healthcare job market demands. Additionally, research on intelligent matching and the construction of knowledge graphs for IE remains limited. This study aims to bridge this gap and alleviate the structural mismatch between the healthcare job market and student employment in China. First, an expertise graph for IE is developed, covering both expertise and healthcare job requirements. A multi-layer fusion information extraction model, combining BERT, BiLSTM, and GCN, is then proposed for knowledge extraction. An employment matching algorithm is introduced to extract healthcare job titles and requirements from the knowledge graph, calculate similarity with students’ overall ability scores, and recommend suitable positions. Finally, a case study demonstrates that the algorithm accurately analyzes students’ ability scores and successfully matches IE majors with relevant healthcare job positions, validating its effectiveness. This study aims to mitigate the structural mismatch between the healthcare job market and student employment, providing high-quality IE talent to medical services, which has significant scientific and practical value.

## 1 Introduction

As higher education expands, the number of college graduates is increasing, with over 11.7 million expected in 2024 (Cheng and Pengfei, 2024). In the context of an economic slowdown, this has intensified pressure on the job market and student recruitment. Concurrently, emerging technologies like digitalization and artificial intelligence (AI) have intensified job market demands for graduate competencies. This has created a skills gap where some graduates fail to meet corporate standards, necessitating dynamic curriculum adjustments in educational institutions (Tiron-Tudor et al., 2025). Meanwhile, students face employment challenges due to information asymmetry between their abilities and job opportunities, suitable positions (Miao and Yuan, 2022). This mismatch creates a structural contradiction between “unfilled jobs” and “unqualified candidates.”

The structural contradictions described are particularly evident in the fields of industrial engineering (IE) and healthcare services. Hospitals, as complex service systems, urgently need to improve operational efficiency through process optimization, resource allocation, and quality management (Mei et al., 2024). However, it is challenging for IE professionals, who possess advanced technical skills and problem-solving abilities, to find suitable opportunities within the complex healthcare system. As a result, IE technology needs to be better applied and adapted to healthcare settings. Moreover, there is an urgent need to establish an intelligent alignment between healthcare job market demands and students’ learning capabilities. This would facilitate optimal human resource allocation, enhance the efficiency and accuracy of recruitment processes, and narrow the gap between graduates’ skills and employers’ needs. Ultimately, this will help reduce the information gap between the healthcare job market and student employment, alleviating the current mismatch between the two.

In order to effectively align these factors and mitigate the structural contradiction, it is essential to systematically and visually correlate the relevant information. Knowledge graph visualization technology, which represents various domain-specific knowledge and their intrinsic relationships in a graph structure (Ling et al., 2021), can play a key role in this context. In particular, expertise graph, a type of vertical knowledge graph, differs from typical knowledge graphs that use knowledge points as nodes. It focuses on the logical relationships between professional disciplines and aims to establish a mapping between the knowledge and competency systems (Jiarong and Shaochun, 2022). This approach is widely used in educational technology. For instance, Caili et al. (2024) developed a knowledge map that integrates course expertise, research, and industry needs to guide course optimization; Guofu et al. (2024) created an industry-academia fusion platform based on this map to enhance mechanical manufacturing and automation courses; Jian et al. (2024) applied environmental and ecological disciplines at University D to construct a framework for planning frontier fields and university development, providing insights for scientific research management. However, current expertise graphs do not adequately capture the intrinsic connection between students’ learning abilities and job market demands. There is also insufficient research on applying multidisciplinary IE knowledge to achieve intelligent matching between student capabilities and medical employment needs, such as hospital process optimization roles.

This paper uses the multidisciplinary field of IE as a case study, constructing a knowledge graph that links professional knowledge with employment demands. It achieves intelligent matching between students’ learning abilities and medical employment needs, offering significant theoretical and practical value in addressing the employment mismatch. This study makes the following key theoretical and practical contributions to the exploration of expertise graph:

1. Existing expertise graph does not address the multidisciplinary field of IE. This paper fills this gap by constructing expertise graph specifically for IE.
2. Existing expertise graphs do not effectively link students’ learning abilities with healthcare job market demands. The IE expertise graph in this paper addresses this gap, establishing a connection and intelligent matching between the two based on disciplinary knowledge.
3. The BERT–BiLSTM–GCN model proposed in this paper can capture multiple feature types, perform feature fusion, and use graph convolution to extract richer structural information. The BERT pre-training model provides semantic features, the BiLSTM captures sequence dependencies, and the GCN uses these enhanced features to model complex entity relationships. The model outperforms traditional methods in baseline data and demonstrates strong algorithmic performance for efficient information extraction.

The TF-IDF-GCN employment matching algorithm proposed in this paper integrates multi-dimensional features for improved ranking and matching, handling sparse data more effectively than traditional collaborative filtering and matrix decomposition approaches, providing more accurate personalized recommendations.

Section 2 reviews the research progress in expertise mapping, knowledge graph construction, and job market demand matching in education. Section 3 presents the process of constructing IE expertise graph, with a focus on an innovative knowledge extraction model based on BERT–BiLSTM–GCN, and develops an algorithm for intelligent matching between students’ learning abilities and job market needs. Section 4 evaluates the reliability and effectiveness of this paper in creating an intelligent match between students’ learning abilities and healthcare job market demands, using actual case studies based on IE expertise graph. Section 5 provides a comprehensive summary and overview of the research findings.

## 2 Literature reviews

Scholars have conducted extensive research on expertise graph in education, methods for constructing knowledge graphs, and the alignment of job market needs.

This study aims to identify whether there is a gap in the construction of expertise graph based on students’ learning abilities in the field of IE, specifically in relation to the needs of the healthcare job market. To address this gap, the paper reviews a wide range of knowledge graph methods and finds that existing knowledge graphs still struggle with handling complex relationships during their construction. As a multidisciplinary field, IE is inherently complex, making it challenging to align it with the evolving needs of the healthcare job market. Therefore, this paper synthesizes current knowledge graph construction methods and proposes a new approach that optimally addresses these complex dependencies. Finally, to enable intelligent matching using the constructed IE expertise graph, research on aligning needs and capabilities must be organized. In summary, this paper focuses on the following three key areas in the literature review.

### 2.1 Expertise graph research within the field of education

Recent research on expertise graph can be broadly categorized into two areas. The first focuses on the extension and optimization of internal connections within the graph. For example, Zhuo et al. (2020) proposed the KQA model of educational knowledge graph, which effectively establishes relationships between knowledge, questions, and abilities. Similarly, Zhu (2024) introduced the KRO model, which strengthens the correlations between knowledge, learning resources, and learning objectives. Shujin and Ruye (2022) developed a method for interdisciplinary knowledge discovery using mapping technology to offer refined knowledge services for researchers and foster scientific research and innovation. Mao and Liping (2022) and Jianghua and Yuliu (2023) both optimized the relationships between disciplinary knowledge by leveraging multimodal resources. The second area addresses the significance and role of mapping in different professional or course domains. For instance, Hongwei (2021) proposed a research framework for constructing knowledge graphs for university online courses, thereby improving course quality. Zhi et al. (2023) tackled issues in traditional education, such as inefficient teaching, lack of personalized instruction, and the heavy burden on both teachers and students. Zuowen (2023) focused on foreign language teaching in universities, associating the field with numerous scattered and unstructured resources. Finally, Zhengqi et al. (2023) proposes a framework integrating a visual disciplinary diversity index and topic modeling, which captures intersections between domains while enhancing the interpretability of cross-disciplinary topic identification results.

In summary, while expertise graph, as a type of vertical knowledge graph, is effective in organizing and optimizing content, teaching resources, and activities within various disciplines such as biology, physics, and foreign languages, existing research does not address the correlation between students’ learning abilities and healthcare job market demands. Furthermore, expertise graph for multidisciplinary fields like IE have yet to be developed in China. Therefore, this paper aims to construct an expertise graph that reflects the relationship between students’ learning abilities and medical employment market needs, considering the complex intersection of multidisciplinary knowledge in IE. This will help alleviate the structural mismatch between the healthcare job market and student employment.

### 2.2 Knowledge graph construction methods research

Knowledge extraction is a crucial first step in graph construction, involving the extraction of entities, attributes, and relationships from various data sources to form an ontological knowledge representation. Common methods for knowledge extraction can be grouped into four main approaches (Yudai et al., 2023; Xu et al., 2023; Ma et al., 2023; Xiao et al., 2023):

1. *Rule-based methods*, which offer high accuracy and interpretability but suffer from high manual effort and low recall.
2. *Deep learning-based methods*, which are highly automated and capable of handling large datasets, but depend on labeled data and still struggle with recall.
3. *Dependency analysis-based approaches*, which capture grammatical structures in sentences but are less effective with long-distance dependencies.
4. *Cross-domain knowledge fusion*, which integrates knowledge from different domains but faces issues such as conflicts, duplication, and complex fusion processes.

Among these, deep learning techniques, especially BERT, BiLSTM, and GCN, have gained widespread use. For example, Wenti et al. (2021) proposed a Local to Global Graph Convolutional Network (LGGCN) to enhance word embeddings by utilizing semantic and structural information from both textual sentences and knowledge graphs. Wei et al. (2022) combined BERT, BiLSTM, and CRF to improve named entity recognition, particularly when labeled data is scarce. Meanwhile, the relative position embedding is further enhanced, and the neural network GeoTPE model is proposed, aiming at the effective extraction of geo-themed phrases from geographic literature (Weirong et al., 2024). Yafei et al. (2023) developed a BERT–BiLSTM–CRF model for citrus pest and disease recognition, creating a specialized corpus to enhance model performance. Nithya et al. (2024) fused BERT-OPCNN and FastText-ACO for rumor detection, addressing false information recognition challenges.

In summary, while significant advancements have been made in fusing deep learning models like BERT and BiLSTM, challenges persist in processing complex relationships, handling task-specific volatility, and improving word embedding quality. To overcome these limitations, this paper proposes an open information extraction model combining BERT, BiLSTM, and GCN. This model will be better suited for handling complex dependency relationships and relational extraction tasks, particularly in the context of mapping the intricate relationships between subject knowledge, students’ learning abilities, and job market requirements.

### 2.3 Job market demands matching research

Research on matching healthcare employment demands with students’ learning abilities remains limited. Warner et al. (2020) examined nursing students’ employability needs and academic performance, finding that factors such as hours worked, work type, and shift schedules significantly affected their academic outcomes (e.g., semester GPA). Jianqiang (2023) analyzed the employability of higher education students in the context of Industry 4.0, using big data methods to identify gaps between students’ skills and employers’ expectations, offering suggestions to improve vocational education and reduce structural unemployment. Wenqi and Changjun (2024) employed statistical and econometric techniques (e.g., regression analysis, chi-square test) to study employment matching for undergraduate graduates, identifying that the mismatch between graduates’ competencies and market needs was a key factor contributing to employment difficulties. Murphy et al. (2025) aligned mental health nursing students’ clinical competencies with healthcare needs. Their exploration of Problem-Based Learning (PBL) in rehabilitation-oriented practice revealed congruence in five elements (such as collaboration, self-directed learning, et al.), with students reporting positive outcomes. Purssell and Sagoo (2025) employed cluster analysis to evaluate nursing education quality diversity in the UK. It found strong nursing employment prospects but weak research-satisfaction correlations and inconsistent institutional performance patterns. The study recommends incorporating broader social value indicators into assessments to bridge theory-practice gaps.

In summary, despite these advances, intelligent matching research between healthcare job demands and student capabilities remains scarce. Consequently, this paper develops a healthcare employment matching algorithm using IE expertise graph to computationally achieve intelligent student-capability-to-healthcare-demand matching.

Based on the insights from the literature review, it is evident that there has been no research focused on constructing expertise graph within the field of IE, nor any studies that leverage such graph to explore the intelligent matching between students’ learning abilities and healthcare job market demands. Therefore, this paper aims to address this gap by developing an intelligent matching system based on IE expertise graph. The objective is to bridge the gap between the healthcare job market and students’ employment prospects, ultimately delivering high-quality IE professionals who meet the evolving needs of medical services in the current era.

## 3 Construction of expertise graph for IE

In this section, we will first focus on the multidisciplinary nature of the IE major as a case study. We will utilize an advanced information extraction model that integrates BERT, BiLSTM, and GCN to construct an IE expertise graph. This expertise graph will serve as a comprehensive knowledge graph, representing key skills, competencies, and concepts within the IE field.

Following the construction of the expertise graph, we designed an employment matching algorithm that intelligently matches students’ learning abilities with the demands of the medical employment market. This paper assesses students’ learning abilities by collecting and evaluating the actual scores of students across each course in the university’s IE syllabus. Each course reflects the students’ learning abilities from different dimensions, and by combining these scores, a comprehensive assessment of the student’s abilities across various dimensions can be made. Then, it extracts healthcare job titles and market requirements from the knowledge graph, calculating the similarity between the healthcare job requirements and the comprehensive ability score of the students. The ability score will be based on various factors, such as the students’ academic performance, technical skills, and other relevant metrics.

Finally, based on this similarity calculation, the system will make intelligent recommendations by sorting the employment positions and suggesting the most suitable job opportunities for each student. These recommendations will ensure a more targeted and effective match between students’ qualifications and the specific needs of the healthcare job market, addressing the gap between education and employment outcomes.

### 3.1 Knowledge acquisition

**Figure 1:**
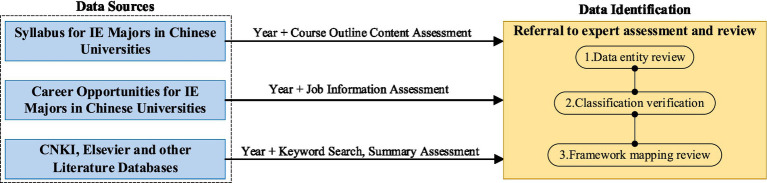
IE related data material selection process.

This paper conducts a thorough data extraction process through deep mining of extensive literature data, drawing from a wide range of sources including current IE training programs and academic research findings, as shown in Figure 1 IE related data material selection process. The data entity review in the expert assessment refers to evaluating the key entities extracted from the data sources; classification verification involves confirming that these entities, initially categorized into 13 defined dimensions, are accurate; and the framework mapping review assesses the accuracy and rationality of these dimensions and the entity data they contain, which have been mapped into the three-tier framework. All of these steps are carried out by senior faculty members with master’s or doctoral degrees or higher, who serve as experts in the field. A total of 59 courses related to the IE discipline, 871 professional papers representing the research contributions of 1,332 scholars and educators, 59 distinct types of employment positions, and 1,321 professional technologies and subject areas closely associated with IE were analyzed.

In addition to the expert assessment, to further validate the subsequent construction of the IE expertise graph, all acquired knowledge resources were compared with the Chinese Occupational Classification Dictionary (COCD) and O*NET. The results aligned with the job descriptions, job skills, and job content for the categories GBM29900 and GBM49900 in the COCD, as well as SOC Code 11–9111.00 and 17–2112.00 in O*NET, without exceeding them.

The research data encompasses several key entity types, which include, but are not limited to: Core courses and subject knowledge forming the professional curriculum system, Students’ academic backgrounds and performance metrics and Academic contributions of renowned scholars and educators in the field, etc. These elements, altogether, form 13 dimensions that describe the full landscape of IE education, expertise, and employment. Referring to the internationally recognized DACUM model (Norton, 2009), these dimensions are categorized into three layers: Expertise Layer (corresponding to the basic curriculum system), Technology Field Layer (corresponding to technical application abilities), and Employment Demand Layer (corresponding to job competencies).

**Figure 2:**
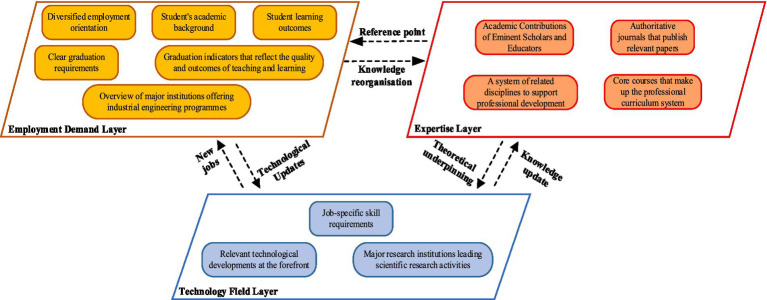
Framework for constructing a mapping of IE expertise.

The interconnections between these layers reflect the relationships between the expertise and the demands of the job market, ultimately forming the IE expertise graph. This framework, as illustrated in Figure 2 highlights how the professional knowledge and skills developed through education are aligned with the needs of the employment market, aiming to improve the intelligent matching between students’ capabilities and job opportunities.

The integration of the expertise layer, technology field layer, and employment demand layer creates a dynamic and interactive framework that enhances the alignment between education and the job market. Together, these three layers create a three-dimensional feedback loop that enables:

1. *Precise focus on professional skills*: Students gain a clearer understanding of the essential skills they need to develop, based on real-world technological trends and job market requirements.
2. *Clear career development orientation*: With comprehensive data, students can more easily identify career paths, job opportunities, and industries that align with their skills and knowledge, guiding them toward informed decisions about their professional futures.

### 3.2 OpenIE model construction

Open Information Extraction (OpenIE) is an automated technique for extracting information that does not rely on predefined rules or frameworks. Its main task is to identify and extract structured triples (object-relationship-object) from text. This technique plays a crucial role in knowledge graph construction and can effectively extract meaningful information from unstructured or semi-structured data. In recent years, deep learning techniques have become widely used in information extraction due to their ability to learn complex semantic patterns and representations from data. This is particularly beneficial for key tasks like named entity recognition (NER), relationship extraction, and entity disambiguation. Various neural network architectures, such as Recurrent Neural Networks (RNN), Convolutional Neural Networks (CNN), and Transformer-based models (e.g., BERT, GPT), have delivered excellent results in these tasks.

#### 3.2.1 BERT–BiLSTM–GCN OpenIE model structure

This paper proposes a multi-layer fusion information extraction model based on BERT, BiLSTM, and GCN, where information flows and deepens across these three modules. This design enables the network to handle a wide range of NLP tasks, especially entity recognition and relationship extraction. Additionally, the model enhances prediction accuracy and generalization ability through customized output and loss function optimization. Specifically, the model uses BERT to preprocess the text and obtain the initial embeddings. Contextual information is then captured by BiLSTM, while node dependencies are modeled using GCN to achieve a rich semantic feature representation.

The input text sequence to the model is denoted as $W=[w_{1},w_{2},w_{3},\ldots ,w_{m}]$, where m represents the sequence length. Initially, BERT is used to generate the base semantic representation of the text sequence through word embeddings. BERT captures contextual dependencies via its multi-layer Transformer structure and produces the embedding vector $E=[e_{1},e_{2},e_{3},\ldots ,e_{i}]$, where $e_{i}$ is the embedding corresponding to the *i*-th word $w_{i}$. Using the pre-trained BERT model, text vectorization is achieved, providing the foundation for subsequent tasks.

**Figure 3:**
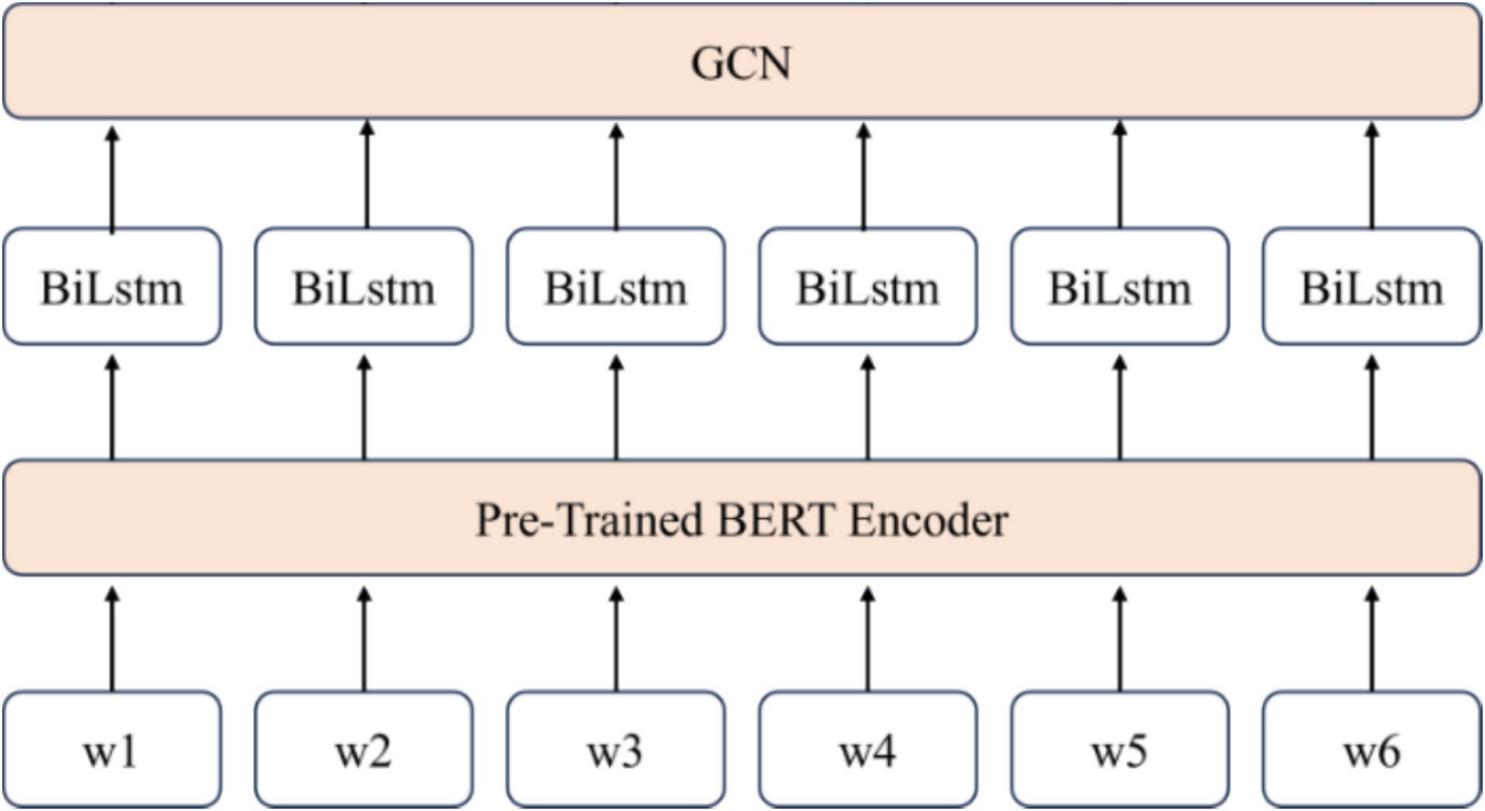
BERT+BiLSTM+GCN open information extraction model.

Next, a BiLSTM network is applied to bi-directionally encode the BERT-generated embeddings, enabling the capture of complete contextual information. The BiLSTM’s bidirectional structure effectively models complex dependencies in the text, enhancing the semantic representation of each word by considering its surrounding context. Finally, the output from the BiLSTM is passed through a GCN layer, which leverages the graph structure to further refine the semantic representations. GCN models node dependencies, enabling the network to better capture entity-to-entity relationships—an essential feature for modeling complex relationships in IE texts. The output from the GCN is then used for entity recognition and relationship extraction tasks. A cross-entropy loss function evaluates the predicted results against the true labels. The model structure is shown in Figure 3 and Pseudo-Code 1, which illustrates the integration of the BERT layer, BiLSTM layer, and GCN layer for public information extraction.

In summary, the BERT–BiLSTM–GCN fusion model presented in this paper effectively captures complex relationships in text by integrating BERT’s deep contextual embeddings, BiLSTM’s bi-directional sequence modeling, and GCN’s graph-based dependency modeling. The model not only excels in accurately identifying named entities but also enhances relationship extraction accuracy through multi-level feature representation, offering comprehensive and rich semantic support for IE information extraction.

#### 3.2.2 Experimental design and analysis of results

To validate the effectiveness of the proposed BERT–BiLSTM–GCN model, an IE dataset was manually labeled and split into training, validation, and test sets in an 8:1:1 ratio. The IE dataset was constructed around education and employment within the IE domain, using raw data from three primary sources, as detailed in Section 3.1 and Figure 1 of the paper. After collecting and integrating the data from these sources, we performed manual annotation to establish a baseline dataset for training and validating our information extraction model (BERT–BiLSTM–GCN). The primary tasks of the annotation process include entity recognition (NER) and relationship extraction. The model’s performance was compared with several other prominent information extraction models, including BERT, BERT–LSTM–CRF, and RoBERTa–CRF:

1. *BERT* is a bi-directional encoder model based on the Transformer architecture. It excels at capturing contextual semantic information and provides high-quality word embeddings that enhance various natural language processing tasks.
2. *BERT–LSTM–CRF* integrates BERT’s contextual embedding capabilities, LSTM’s sequence modeling power, and CRF’s sequence label constraints. This combination makes it highly effective in named entity recognition (NER) tasks through its multi-layer architecture.
3. *RoBERTa–CRF* is an enhanced version of BERT, where the RoBERTa model is used instead of BERT. It also incorporates a CRF layer to further improve text semantic understanding and enhance sequence annotation accuracy for classification and information extraction tasks.

The evaluation of all models was based on Precision, Recall, and F1 score, as defined by the following Equations 1–3:


(1) $$\text{Precision}=\frac{\mathrm{TP}}{\mathrm{TP}+\mathrm{FP}}$$

(2) $$\text{Recall}=\frac{\mathrm{TP}}{\mathrm{TP}+\mathrm{FN}}$$

(3) $$F1-\text{score}=\frac{2\mathrm{TP}}{2\mathrm{TP}+\mathrm{FP}+\mathrm{FN}}$$

Where TP, FP and FN denote the number of true positive, false positive and false negative samples, respectively. These evaluation metrics provide a comprehensive measure of the model’s ability to perform information extraction tasks, highlighting its strengths in correctly identifying relevant entities and relationships while minimizing errors.

The experimental results, as shown in Table 1, indicate that the BERT–BiLSTM–GCN model proposed in this paper outperforms the comparison models in terms of precision, recall, and F1 scores for the task of information extraction within the IE domain. The key factor contributing to this model’s superior performance is the GCN’s role in relation extraction, which enhances its ability to capture and model the structural relationships within the text.

**Table 1:** Results of model performance comparison.

Model name	P	R	F1-score
BERT	0.9543	0.9543	0.9542
BERT–LSTM–CRF	0.9591	0.9589	0.9587
RoBERT–CRF	0.7023	0.7023	0.7023
BERT–BiLSTM–GCN	0.9617	0.9613	0.9613

Overall, to address the structural mismatch between IE graduates and healthcare job requirements, this study employed a multi-model fusion approach comprising BERT, BiLSTM, and GCN for knowledge extraction. A deeper analysis of the model performance differences reveals that each component contributes uniquely to the final outcome. BERT demonstrates superior capability in understanding the contextual semantics of healthcare job descriptions, which often contain complex phrases, domain-specific terminology, and implicit requirements. Its attention mechanism enables it to capture long-range dependencies and nuanced meanings within the text, which is particularly valuable in interpreting unstructured job descriptions. However, BERT alone does not model the relational structure among entities—such as the associations between skills, academic courses, and job roles—which limits its effectiveness in structural reasoning tasks.

In contrast, BiLSTM exhibits strength in capturing local sequential patterns, such as ordered lists of qualifications or skill sequences in resumes. Its lightweight architecture contributes to faster training and better generalization on limited datasets. Nonetheless, BiLSTM lacks the depth of contextual understanding present in transformer-based models, and its performance degrades with increasing text length, which is common in IE curriculum descriptions and job postings. GCN, on the other hand, excels in learning from the topological structure of the constructed expertise graph. It effectively models the connections among educational content, student capabilities, and job requirements, making it particularly useful in domain-specific relation extraction and graph reasoning. Yet, its performance is highly sensitive to the quality of the input graph and is limited when node feature representations are insufficiently expressive. The combined BERT–BiLSTM–GCN architecture leverages the strengths of all three models: BERT for semantic richness, BiLSTM for sequential structure, and GCN for graph-based relational inference. This hybrid model not only improves entity recognition and relation extraction accuracy but also enhances the interpretability and robustness of employment recommendation results.

We selected a complex sentence containing multiple immediately neighboring entities from the new dataset for simulation prediction to compare the boundary recognition ability of different models. Test sentence: “Lead or participate in quality improvement projects such as FMEA, Six Sigma, etc.” Ground Truth: [Skills: FMEA], [Skills: Six Sigma], [Responsibilities: Quality Improvement]. Examples of test data are shown in Table 2 Example of test data.

**Table 2:** Example of test data.

Model name	Simulation of forecast results
BERT (base line)	[Skills: FMEA, Six Sigma] [Responsibilities: Quality Improvement Projects]
BERT–LSTM–CRF	[Skills: FMEA], [Skills: Six Sigma] [Responsibilities: Quality Improvement Projects]
RoBERT–CRF	[Skills: FMEA] [Responsibilities: Quality Improvement]
BERT–BiLSTM–GCN	[Skills: FMEA], [Skills: Six Sigma] [Responsibilities: Quality Improvement]

In summary, the experimental results show that the BERT–BiLSTM–GCN model proposed in this paper outperforms other mainstream methods in the information extraction task, particularly in Precision, Recall, and F1 score, with significant improvements. The model’s efficient performance in course feature extraction and career matching highlights its practical advantages and effectiveness. These results underscore the model’s potential for knowledge graph construction and information retrieval, providing valuable insights and a solid foundation for further research in these areas.

### 3.3 Ontology construction of the IE

Building on the BERT–BiLSTM–GCN open information extraction model, information extraction is performed on the collected research data, leading to the construction of an ontology for the IE major. This paper primarily focuses on the degree stage of IE, incorporating academic contributions from renowned scholars and educators. The research emphasizes employment orientation, core skills, and draws on relevant data from major higher education institutions.

The extracted data is systematically organized into multiple ontology levels, with categories including Majors, Employment Opportunities, Institutions of Higher Learning, Academic Degrees, Courses, and Academic Papers.

**Figure 4:**
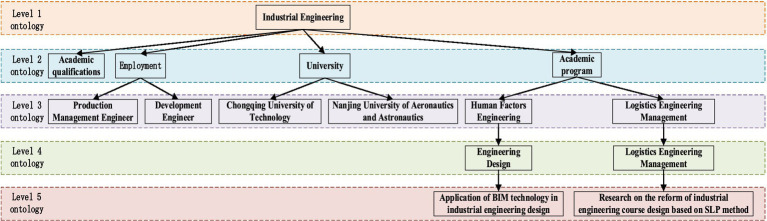
IE ontology design.

This hierarchical structure provides a more comprehensive view of the IE field, ensuring that all critical aspects, from education to career prospects, are addressed. The constructed ontology, as depicted in Figure 4, allows for a detailed and organized representation of the IE domain, aiding in a deeper understanding of the relationships between various elements in this field.

### 3.4 IE expertise graph storage and visualization

**Figure 5:**
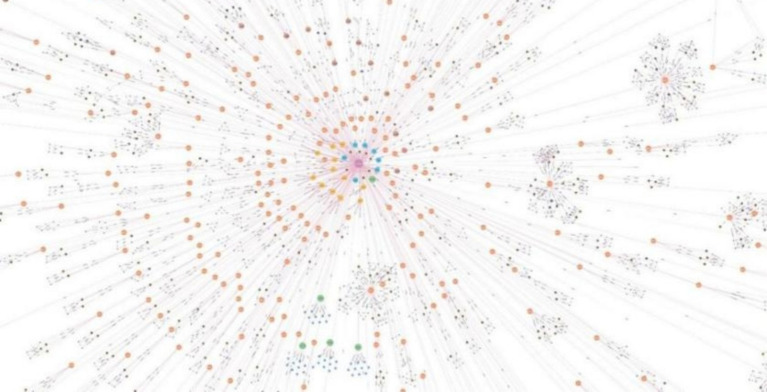
Mapping of IE expertise.

In this paper, we utilize the Neo4j graph database for both data storage and visualization. Based on the ontology design presented in Figure 4, we successfully constructed an IE expertise graph. This graph contains 4,851 entities and 11,157 relationships, as illustrated in Figure 5.

### 3.5 Knowledge reasoning methodology construction

The graph structure of a Knowledge Graph effectively represents the interdependence between courses and their connections to careers, offering an intuitive perspective for understanding the interactions within the educational domain. This paper utilizes TF-IDF vectors to analyze course content characteristics and incorporates additional markers, such as whether the course is a core subject or its difficulty, to create a multi-dimensional course description.

#### 3.5.1 Knowledge reasoning methodology design

This paper uses a GCN to learn the embedded representation of each node in the graph. The advantage of GCN is its ability to fully leverage the graph topology, allowing each node’s representation to incorporate both its own features and information from neighboring nodes, thereby enhancing the richness and accuracy of the representation. For similarity computation, this paper proposes a dual similarity strategy (see Pseudo-Code 2), consisting of two main modules:

(1) *GCN similarity*: The cosine similarity between the candidate’s course embeddings and the node embeddings of each occupation is computed using the trained GCN model to evaluate the candidate’s relevance to the occupation.

(4) $$S_{\mathrm{GCN}}(c,j)=\cos(e_{c},e_{j})$$

In Equation 4, $e_{c}$ is the embedding vector of candidates, and $e_{j}$ is the embedding vector of job.

(2) *Course similarity*: A weighted course similarity score is calculated based on the match between the candidate’s course grades and the courses required for the career, reflecting the fit between the candidate’s academic background and the career’s requirements.

(5) $$S_{\text{course}}(c,j)=\sum_{k\in \text{required}\_\text{course}(j)}\cos(e_{c},e_{k})$$

In Equation 5, $e_{c}$ is the embedded representation of the candidate’s courses, and $e_{k}$ is the embedded representation of the courses required for the job.

Additionally, a course weight adjustment mechanism is introduced in the similarity calculation to emphasize the importance of highly career-related courses by increasing their weight in career matching. Simultaneously, higher weights are assigned to core major courses to reflect their centrality in the educational context.


(6) $$S(c,j)=\alpha \cdot S_{\mathrm{GCN}}(c,j)+\beta \cdot S_{\text{course}}(c,j)$$

(7) $$\max_{c,j}S(c,j)$$

In Equation 6, α and β are hyperparameters controlling the contributions of GCN similarity and course similarity to the final matching score. In this study, both parameters are set to 0.5, indicating that the weight on both sides have equal components. The objective of this model, as shown in Equation 7, is to maximize the matching score between candidates and careers, recommending the most suitable career for each candidate.

#### 3.5.2 Experimental performance comparison

As shown in Equations 8–10, this paper evaluates the effectiveness of the algorithm using metrics such as the confusion matrix and NDCG@5. Specifically, ${\mathrm{rel}}_{i}$ denotes the true relevance score of the *i*-th result, ∣REL∣ represents the set of true relevance scores sorted in descending order, and the number of sets refers to the first K results.


(8) $$\text{Precision}@5=\frac{\mathrm{TP}@5}{\mathrm{TP}@5+\mathrm{FP}@5}$$

(9) $$\text{Recall}@5=\frac{\mathrm{TP}@5}{\mathrm{TP}@5+\mathrm{FN}@5}$$

(10) $$\begin{array}{l} \text{NDCG}@5=\frac{\mathrm{DCG}@5}{\text{IDCG}@5},\mathrm{DCG}@5=\sum \frac{{\mathrm{rel}}_{i}}{\log(i+1)},\\ \text{IDCG}@5=\sum_{i=1}^{|\mathrm{REL}|}\frac{{\mathrm{rel}}_{i}}{\log(i+1)} \end{array}$$

As shown in Table 3, the model proposed in this paper performs well across all three evaluation metrics, demonstrating strong recommendation results. Specifically, the algorithm achieves scores of 0.6667, 0.6667, and 0.6164 for precision@5, recall@5, and NDCG@5, respectively, which are significantly better than those of the other compared methods. In contrast, the collaborative filtering model yields scores of 0.333, 0.5, and 0.4704; the content-based recommendation method achieves scores of 0.6667, 0.5, and 0.2773; and the cosine similarity approach results in 0.6, 1, and 0.4913. These results demonstrate that the proposed algorithm is more effective in satisfying recommendation needs by improving information retrieval accuracy and enhancing user satisfaction.

**Table 3:** Comparison of similarity algorithm metrics.

Model name	P@5	R@5	NDCG@5
Collaborative filtering	0.333	0.5	0.4704
Content-based recommendations	0.6667	0.5	0.2773
cosine similarity	0.6	1	0.4913
Double similarity calculation strategy	0.6667	0.6667	0.6164

## 4 Case study

IE, as an interdisciplinary domain integrating management science, engineering, and economics, necessitates tailored skill sets aligned with specific occupational demands. Divergent core competencies emerge across roles: manufacturing engineers require lean production implementation and process optimization expertise, whereas systems analysts demand advanced proficiency in modeling, simulation, and data-driven optimization.

This section validates the proposed algorithm by empirically analyzing two major job roles within the healthcare services domain that are closely linked to IE technologies: Hospital Process Optimization Engineer (HPOE) and Medical Logistics Analyst (MLA). By analyzing the official job employment requirements posted online and cross-referencing them with the employment descriptions and requirements of the corresponding job categories in COCD and O*NET, we have compiled the job descriptions and key skills, as shown in Table 4. The study demonstrates how the expertise graph and the employment matching algorithm enable precise alignment between students’ learning abilities and sector-specific demands.

**Table 4:** IE Job Demand Extraction.

Job title	Academic requirements	Job descriptions	Sampling results
HPOE	Undergraduate or higher	1. Leading the digital transformation of core hospital processes (including outpatient triage systems, operating room scheduling solutions, emergency response mechanisms); 2. Reduced average patient wait time by ≥25% by implementing clinical pathway optimization through PDCA cycle; 3. Modeling of healthcare resource projections (bed turnover/medical care ratios/equipment utilization) using discrete event simulation techniques; 4. Coordination of cross-sectoral implementation of process re-engineering projects under DRG/DIP payment reforms.	Job title: HPOE, key skills: healthcare process optimization, process improvement, quality control, project management.
MLA		1. Design of a full traceability system for the cold chain of pharmaceuticals (2–8 °C temperature control compliance rate ≥99.9%); 2. Establishment of an intelligent storage model for high-value consumables, realizing interoperative delivery time of ≤15 min; 3. Optimized path planning for hospital logistics robots to reduce in-hospital transportation costs by 30%+; 4. Building a medical emergency logistics response system.	Job title: MLA, key skills: logistics management system, logistics routing, material quality control, project management.
Job requirements		Bachelor’s degree in IE, biomedical engineering or healthcare management, good communication skills, analytical thinking, strategic thinking to prioritize tasks, good organizational skills.	

In Table 4, “IE Job Demand Extraction,” this study examines two roles within healthcare delivery that are closely associated with IE technology: HPOE and MLA, algorithmically extracting their core skill sets. For HPOE, the essential competencies revolve around four key areas: Healthcare process optimization, process innovation, quality control, and project management. For MLA, the focus shifts to skills in establishing and improving logistics management system, logistics routing, material quality control, and project management.

The competency requirements for IE graduates vary significantly across different positions. As shown in Table 4, HPOE focus on re-engineering clinical pathways and digital transformation of hospital operations. This role requires expertise in healthcare systems simulation, multi-stakeholder coordination across clinical departments, and proficiency in patient flow analytics. Conversely, MLA prioritize precision management of pharmaceutical supply chains and mission-critical medical resource allocation. This position demands specialized knowledge in cold-chain integrity protocols and regulatory compliance within stringent healthcare safety frameworks.

In Table 5, “Empirical Analysis of Employability Matching in IE,” the healthcare employability fitness of two students, Li Ming and Yang Liang, is analyzed in detail. An employment matching algorithm calculates the degree of match between each candidate and their target occupation, with ratings as follows: A+ for a match of 95 or more, A for 90–95, B+ for 85–90, and so on.

**Table 5:** Empirical analysis of employability matching in IE.

*Li Ming*’s overall ability score	*Yang Liang*’s overall ability score
Introduction to IE: 92 Production planning and control: 98 Introduction to human factors engineering: 92 Engineering basics: 87 Quality control and management: 85 Project management and coordination: 95	Introduction to IE: 95 Production planning and control: 78 Introduction to human factors engineering: 85 Engineering basics: 96 Quality control and management: 96 Project management and coordination: 82

Findings of the analysis							
	Job title	Process optimization	Quality management system	Process improvement	Quality control	Project management	**Comprehensive rating**
Li Ming	HPOE	A+	—	A	B	A+	**A**
	MLA	—	B+	B	B	A+	**B+**
Yang Liang	HPOE	C+	—	A	A	B	**B−**
	MLA	—	A+	A	A	B−	**A**

Li Ming demonstrated a strong professional foundation, scoring 98 points in production planning and control. His solid performance in the Fundamentals of Engineering and Introduction to Engineering further reinforced his fit for process optimization, project management and process improvement, earning him an A+ and A grade. Although his scores in Quality Control was somewhat lower, maintaining a B grade at the mid-to-high range still provided him with a competitive advantage for the HPOE position, resulting in an overall A grade.

On the other hand, Yang Liang scored 96 points in the Engineering Fundamentals Assessment and earned excellent A grades in both Process Improvement and Quality Control, showcasing his potential for roles in quality control and process innovation. This made him a perfect fit for the MLA position, earning him a high A grade overall. However, in the HPOE role, despite strong performance in Process Improvement and Quality Control, his score of 78 points in the critical area of Production Planning and Control resulted in a C+ grade for production process optimization. This lowered his overall assessment to a final grade of B−.

In summary, the employment matching algorithm developed in this study effectively facilitates personalized, intelligent matching between IE students and job positions. By accurately analyzing students’ academic performance, the algorithm emphasizes the scientific and practical importance of guiding students to select careers that align with their academic strengths. Additionally, it offers valuable insights and a reference framework for assessing employability and shaping the orientation and training of IE professionals.

## 5 Conclusion

This study addresses the gap in intelligent matching research related to the alignment between students’ learning abilities and healthcare job market demand, particularly within the field of IE. It aims to mitigate the structural imbalances in the domestic employment market by leveraging expertise graph in IE.

The research process is structured as follows:

1. *IE Expertise Graph Construction*: The study first develops a comprehensive expertise graph for IE. This graph captures the essential knowledge and skills needed in the field, providing a structured representation of the discipline’s core competencies.
2. *Multi-layer Fusion Information Extraction Model*: A sophisticated information extraction model is proposed, combining BERT, BiLSTM, and GCN. This model effectively extracts relevant knowledge from textual data, enabling deeper insights into both students’ abilities and the job market’s requirements.
3. *Employment Matching Algorithm*: The study designs an employment matching algorithm that uses the knowledge graph to extract healthcare job titles and their associated requirements. The algorithm calculates a double similarity score—comparing students’ comprehensive ability scores with healthcare job demands—thus identifying the most suitable employment positions for each student.
4. *Evaluation through Case Studies*: The effectiveness and accuracy of the algorithm are validated through practical case studies, demonstrating its potential to align students’ learning profiles with appropriate career opportunities.

Overall, this study offers a novel approach to bridging the gap between academic training and employment needs, with a particular focus on IE, helping to solve the mismatch problem in the healthcare job market. Case studies confirmed that the fusion model outperforms any single architecture in matching IE graduates to suitable healthcare job roles, especially in scenarios requiring both semantic and structural understanding. Future work may further explore transformer-based graph neural networks or knowledge distillation techniques to compress the model and improve scalability for real-world applications in university career counseling systems. However, the current three-tier framework for mapping industrial engineering expertise relies solely on qualitative analysis, lacking quantitative methods to better illustrate the strength of connections between the tiers. Additionally, using TF-IDF to compute course feature vectors for job matching may lead to a poor match when course names do not precisely correspond to the required skills. Moreover, the dual similarity strategy in this study does not account for specific feature differences between positions and candidates, limiting its adaptability to all scenarios. Future research will integrate quantitative analysis to refine the connections within the three-tier industrial engineering expertise mapping framework. It will also incorporate more contextual information, personalized features of candidates and positions, and introducing appropriate feedback mechanisms to improve matching quality.

## Data Availability

The raw data supporting the conclusions of this article will be made available by the authors, without undue reservation.
