# Supplementary material for: Research on intelligent matching of students’ learning ability and healthcare job market demand based on industrial engineering expertise graph
Source: Front Artif Intell. 2025 Sep 12;8:1650095. doi: 10.3389/frai.2025.1650095 (PMC12463903; doi:10.3389/frai.2025.1650095)
Supplement: Supplementary file 1 [file Supplementary_file_1.docx]

**Appendix**

| **Pseudo-Code 1**: BERT - BILSTM - GCN model for entity recognition |
| --- |
| #Import necessary libraries  import torch  import torch.nn as nn  import torch.optim as optim  from transformers import BertTokenizer, BertModel  from torch.utils.data import DataLoader  from torch.nn.utils.rnn import pad_sequence  from torchcrf import CRF    # Set device  device = torch.device("cuda" if torch.cuda.is_available() else "cpu")    # Define custom dataset  class NERDataset(torch.utils.data.Dataset):  def __init__(self, texts, labels):  self.texts = texts  self.labels = labels  self.tokenizer = BertTokenizer.from_pretrained("bert-base-uncased")    def __len__(self):  return len(self.texts)  # NER model  class NERModel(nn.Module):  def __init__(self, bert_model, BILSTM_model， gcn_layers, num_labels):  super(NERModel, self).__init__()  self.bert_model = bert_model  self.BILSTM_model = BILSTM_model  self.gcn_layers = gcn_layers  self.fc = nn.Linear(gcn_layers[-1], num_labels)  self.crf = CRF(num_labels, batch_first=True)    def forward(self, input_ids, attention_mask, adjacency_matrix):  bert_output=self.bert_model(input_ids=input_ids, attention_mask=attention_mask)[0]  blstm_input = bert_output  blstm_output = blstm_input  for gcn_layer in self.gcn_layers:  gcn_output = gcn_layer(blstm_output, adjacency_matrix)    out = self.crf (gcn_output)  return out  # Set hyperparameters and initialize the model  num_labels = 10 # Number of labels  bert_model = BertModel.from_pretrained("bert-base-uncased") # Pre-trained Bert lstm_out, hidden = nn.LSTM(bert_embedding,  self.hidden_dim,  num_layers=self.rnn_layers,  bidirectional=True,  batch_first=True)  gcn_layers = [GCNLayer(in_features= lstm_out, out_features=64),  GCNLayer(in_features=64, out_features=64)]  model = NERModel(bert_model, BILSTM_model， gcn_layers, num_labels).to(device)    # Define loss function and optimizer  criterion = nn.CrossEntropyLoss()  optimizer = optim.Adam(model.parameters(), lr=0.001)    # Preprocessing step: Tokenize and encode the texts  texts = [...] # List of input texts  labels = [...] # List of corresponding labels  dataset = NERDataset(texts, labels)    # Create data loader for batch processing  batch_size = 16  dataloader = DataLoader(dataset, batch_size=batch_size, shuffle=True, collate_fn=collate_fn)    # Define training loop  for epoch in range(num_epochs):  # Iterate over training data  for input_ids, attention_mask, graph, labels in dataloader:  # Forward pass through the model to get predictions  predictions = model.forward(input_ids, attention_mask, graph)    # Compute loss based on predictions and ground truth labels  loss = compute_loss(predictions, labels)    # Backpropagation and optimization step  optimizer.zero_grad()  loss.backward()  optimizer.step()  # Evaluate model performance on validation set  accuracy = evaluate(model, validation_data)  print("Epoch:", epoch, "Accuracy:", accuracy)  # Save the trained model for future use  model.save_model() |

| **Pseudo Code 2**: Employment Matching |
| --- |
| import numpy as np import torch import torch.nn.functional as F from torch_geometric.nn import GCNConv import networkx as nx from py2neo import Graph from sklearn.feature_extraction.text import TfidfVectorizer from sklearn.metrics.pairwise import cosine_similarity  # Neo4j connection configuration NEO4J_URI = " " NEO4J_USER = " " NEO4J_PASSWORD = " "  # Retrieve data def read_data_from_graph_db():  graph = Graph(NEO4J_URI, auth=(NEO4J_USER, NEO4J_PASSWORD))  nodes_query = "MATCH (n) RETURN id(n) AS id, labels(n) AS type, n.name AS name"  edges_query = "MATCH (a)-[r:RELATED]->(b) RETURN id(a) AS source, id(b) AS target, r.weight AS weight"  nodes = list(graph.run(nodes_query))  edges = list(graph.run(edges_query))  return nodes, edges  # Compute the TF-IDF vector  def calculate_tfidf_vectors(courses):  # Compute the TF-IDF vector and return  return tfidf_matrix  # Creating diagram structures  def create_graph(nodes, edges, tfidf_vectors):  G = nx.Graph()  for node in nodes:  features = tfidf_vectors[node['id']] if 'course' in node['type'] else [0] * len(tfidf_vectors[0])  G.add_node(node['id'], features=features, type=node['type'], name=node['name'])  for edge in edges:  G.add_edge(edge['source'], edge['target'], weight=edge['weight'])  return G  # Preparing GCN inputs def prepare_gcn_input(G):  A = nx.adjacency_matrix(G)  X = torch.FloatTensor([G.nodes[node]['features'] for node in G.nodes()])  return torch.FloatTensor(A.todense()), X  # GCN model class GCN(torch.nn.Module):  def __init__(*self*, input_dim, hidden_dim, output_dim):  super(GCN, *self*).__init__()  *self*.conv1 = GCNConv(input_dim, hidden_dim)  *self*.conv2 = GCNConv(hidden_dim, output_dim)   def forward(*self*, x, edge_index):  edge_index = edge_index.nonzero().t().contiguous()  x = F.relu(*self*.conv1(x, edge_index))  x = *self*.conv2(x, edge_index)  return x  def improved_job_matching(candidate, knowledge_graph, trained_model, G):  job_scores = {}  # Get an embedded representation of the candidate  candidate_embedding = get_candidate_embedding(candidate, trained_model, G)    for job, required_courses in knowledge_graph.items():  # Get an embedded representation of the work  job_embedding = get_job_embedding(job, trained_model, G)    # Calculating GCN similarity  gcn_similarity = calculate_gcn_similarity(candidate_embedding, job_embedding)    # Calculate the course similarity score  course_similarity_score = calculate_course_similarity(candidate, required_courses, trained_model, G)    # Combining GCN similarity and course similarity scores  combined_score = combine_scores(gcn_similarity, course_similarity_score)    job_scores[job] = combined_score    # Sort jobs and return results  return sort_jobs(job_scores)  # Helper function  def get_candidate_embedding(candidate, model, G):  # Get an embedded representation of the candidate  return G.nodes[candidate['id']]['features']  def get_job_embedding(job, model, G):  # Get an embedded representation of the work  return G.nodes[job]['features']  def calculate_gcn_similarity(embedding1, embedding2):  # Calculate the similarity between two embeddings  return F.cosine_similarity(embedding1, embedding2).item()  def calculate_course_similarity(candidate, required_courses, model, G):  # Calculate the similarity score between the candidate's program and the required program  total_score = 0  for course in required_courses:  course_embedding = G.nodes[course]['features']  total_score += calculate_gcn_similarity(candidate['embedding'], course_embedding)  return total_score  def combine_scores(gcn_score, course_score):  # Combining GCN scores and course scores  return 0.5 * gcn_score + 0.5 * course_score  def sort_jobs(job_scores):  # Sorting the work  return sorted(job_scores.items(), key=lambda x: x[1], reverse=True) |
